# ‘Labour Hopscotch’: Women’s evaluation of using the steps during labor

**DOI:** 10.18332/ejm/152492

**Published:** 2022-09-09

**Authors:** Lorraine Carroll, Sinead Thompson, Barbara Coughlan, Teresa McCreery, Aisling Murphy, Jean Doherty, Lucille Sheehy, Martina Cronin, Mary Brosnan, Denise O’Brien

**Affiliations:** 1 School of Nursing, Midwifery and Health Systems, University College Dublin, Dublin, Ireland; 2 National Women and Infants Health Programme, Dublin, Ireland; 3 National Maternity Hospital, Dublin, Ireland

**Keywords:** normal birth, midwifery, models of care, maternity research, innovation, active birth

## Abstract

**INTRODUCTION:**

Concerns have been expressed globally about the decline in rates of physiological birth and rising intervention rates during labor and birth. The ‘Labour Hopscotch’ Framework, a visual depiction of steps required to remain active during labor was implemented in a large tertiary maternity hospital in Ireland. The aim of this study was to evaluate the steps of the Labour Hopscotch women found most useful, examine the use of non-pharmacological and pharmacological methods of pain relief used during labor and finally to investigate the labor and birth outcomes of women who used ‘Labour Hopscotch’ during labor.

**METHODS:**

A descriptive cross-sectional study was conducted using a study specific questionnaire.

**RESULTS:**

A total of 809 women completed the questionnaire. The Labour Hopscotch Framework was positively evaluated. Mobilizing, the birthing ball, birthing stool, and water therapy were found to be the most useful steps. Primiparous women were more likely to use non-pharmacological methods of pain relief. Pharmacological methods used by women were entonox (67.5%), pethidine (8%) and epidural analgesia (38.5%). Primiparous women were more likely to have epidural analgesia than multiparous women (p<0.00001). Women that attended either private (p=0.004) or public-led obstetric (p=0.005) antenatal care were more likely to have epidural analgesia in labor. Women attending the community midwives were least likely to receive epidural analgesia during labor. The rates of spontaneous vaginal birth, assisted birth and cesarean section, were 77.1%, 14% and 8.7%, respectively.

**CONCLUSIONS:**

Our study findings contribute to the increasing national and international evidence that initiatives such as Labour Hopscotch can promote and advocate for women to be active and mobile during labor to support physiological birth.

## INTRODUCTION

Advances in medical technologies and interventions can provide significant benefits for maternal and infant health, especially in high-risk pregnancies and premature births. In some countries, the use of obstetric interventions in labor and birth has become the norm. It is known that unnecessary interventions such as continuous electronic fetal monitoring^1^, amniotomy^2^ and epidural analgesia^3,4^ undertaken without indication during labor, can disrupt the natural physiology and progression of labor, thus increasing the risk of cascades of further interventions including instrumental birth or cesarean section. Concerns have been expressed globally about the decline in the rate of physiological birth and rising intervention rates during labor and birth^5,6^. Worldwide calls have been made that the use of interventions should be based on evidence that enhances maternal and infant health, with more emphasis placed on supporting physiological processes^7,8^. A drive for normal physiological birth has subsequently resurged in recent years with growing support from the publication of numerous studies demonstrating the benefits of midwifery-led care^9^, continuous one-to-one intrapartum support^4,10^, mobilization and optimal positioning during labor^11,12^ in reducing the cascade of further interventions during labor and birth.

Despite global trends of declining birth-rates, Ireland continues to have one of the highest birth-rates in Europe. Just over 60000 babies are born each year across nineteen maternity units and hospitals. Maternity care is provided through either free public, fee-based private or semi-private obstetric-led services; the majority of births occur in the hospital setting, with less than 1% of all births occurring at home^13^. Women can also avail themselves of antenatal midwifery-led clinics or integrated hospital community midwifery care if available in some regions of Ireland. In Ireland, the rate of cesarean section is currently 35.4%^14^ and expected to continuously increase. The national rate for epidural analgesia is 41.6%^14^.

**Figure 1:**
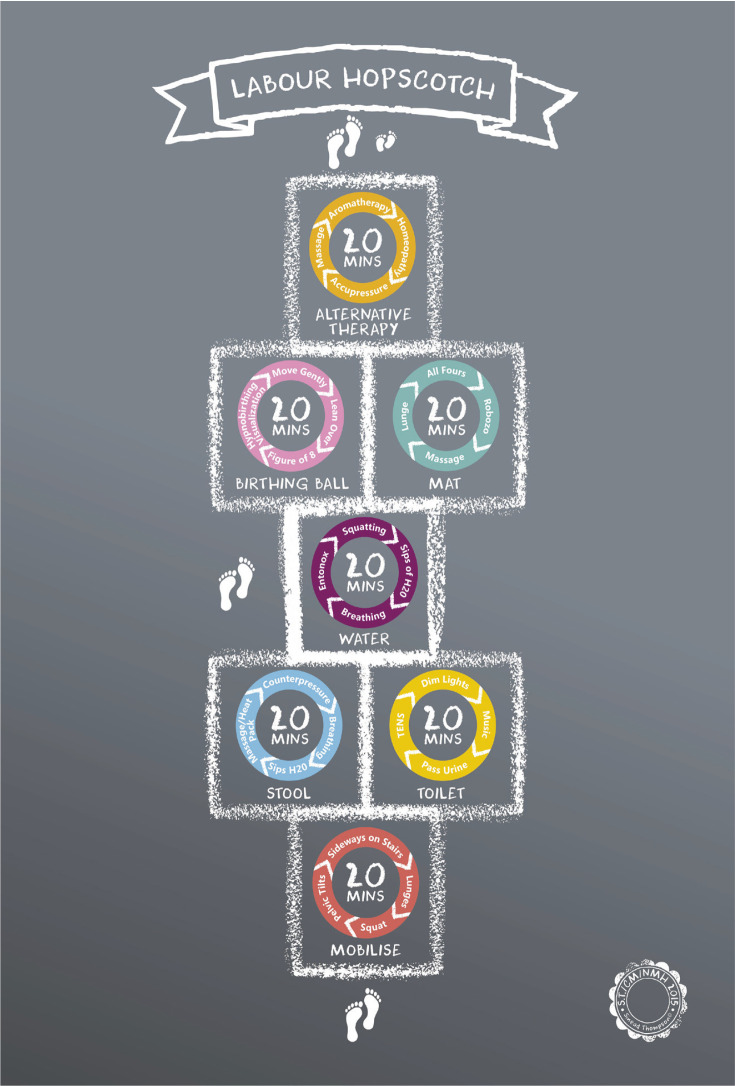
The Labour Hopscotch Framework

This study arose following a midwifery normal birth forum held in a large urban maternity hospital in the Republic of Ireland where concerns were raised about the increasing rates of epidural analgesia use amongst women. Nearly 60% of the total hospital population giving birth had epidural analgesia during labor; and 68.4% for nulliparous women, a concerning rate. A discussion arose about developing and introducing evidence-based innovations that could reduce the rate of interventions and facilitate normal physiological birth for women. In 2015, a community midwife designed and produced a visual framework called the ‘Labour Hopscotch’ (Figure 1). The framework was designed as a tool that can be used by women while being supported by their birthing partner during labor and birth. In addition, the framework was also developed to support midwives as a means of supporting women to achieve a physiological birth. The fundamental principle of the Labour Hopscotch is to inform both women and midwives of the importance of the steps/measures necessary to remain active during labor, thus promoting optimal fetal positioning, which is vital to achieve a physiological birth. These steps include the use of mobilization, positioning, hydrotherapy and non-pharmacological methods of pain relief. An appropriate time frame is provided for each step and is illustrated in a sequential manner that is matched with the progression of labor. Women start at the bottom of the hopscotch which represents early labor when the woman is usually more active and mobile. As labor progresses, the woman advances through the steps of the Labour Hopscotch towards the baby’s footprints. This is a motivational image for women to visualize and facilitates women to maintain focus during labor.

The ‘Labour Hopscotch’ framework (LHF) innovation formed part of an initiative to promote normal physiological birth at the maternity hospital. The aim of this study was to determine the most common steps of the LHF used by women during labor, which steps women found most helpful, and to examine labor and birth outcomes such as pain relief used and mode of birth in women who used the LHF during pregnancy and labor. This article provides results from a cross-sectional study. The experiences of using the LHF from the perspective of women and their partners^15^ and midwives^16^ are reported elsewhere.

## METHODS

### Study design, setting and sample

A cross-sectional descriptive study, using a study-specific questionnaire, was conducted. Women who were attending a large urban tertiary referral maternity unit in the Republic of Ireland (ROI) were invited to complete the questionnaire. The hospital site is one of the largest maternity hospitals in Europe with nearly 8000 births per year, accounting for nearly 13% of all births in the Republic of Ireland. Antenatal care is provided through obstetric-led clinics (public, semi-private and private), midwife-led clinics and other midwife-led schemes that provide Homebirth, DOMINO (a midwife-led maternity service, antenatal visits take place in the hospital or community setting, the woman births in hospital and can be discharged home after six hours of birth) and Early Transfer Home services (the woman births in hospital and discharged home after twelve hours post vaginal birth or on day four following a cesarean section). Hospital labor ward midwives or community midwives provide care to all women during labor and birth, usually on a one-to-one basis; obstetricians are not present for labor or births unless complications occur, or the woman has opted for fee-based private care. Active management of labor is a philosophy of care that is practiced within the hospital that begins with antenatal preparation classes^17^. This approach to care may differ from other approaches to maternity care available in the ROI and includes a strict criterion for the diagnosis of labor, early amniotomy, early use of oxytocin for poor progress in labor, and one-to-one midwifery care. Alternative complementary therapies are not provided at the clinic site, therefore women who want to use them must arrange for these supports themselves. At the time of data collection, water immersion in a birthing pool was only available as a method of pain relief to women who opted for a homebirth with the community midwifery service.

### Inclusion and exclusion criteria

Women were eligible to take part in the study if they had a singleton, healthy uncomplicated pregnancy, were aged ≥18 years, had sufficient English to complete the questionnaire, and had used the steps of the LHF during labor. Women were excluded from the study if they were aged <18 years, if their English was inadequate, could not give informed consent, had a multiple pregnancy, and had experienced a miscarriage, stillbirth or neonatal death.

### Sample size calculation

Based on the monthly birth numbers of the participating hospital site, it was calculated that to obtain a sufficient representative sample, the questionnaire should be conducted over a 3-month period with a target population of 2400 women. Based on responses to the pilot study, the response rate was anticipated to be as high as 70%. The expected epidural rate of 70% was estimated with a precision (95% confidence interval width) of ±2.6%. The worst-case precision with a sample of this size would be ±2.8%. A sample size of 800 was set as the target for this phase of the study.

### Recruitment

Women attending the hospital were informed of the LHF study by midwives during antenatal education classes and during antenatal visits. Visual images of the framework were displayed in outpatient departments and the antenatal ward of the hospital. Samples were also readily available for women to take home and it was also free to download via the hospital’s webpage. Women were provided with an opportunity to read an information leaflet about the study during their pregnancy. Participants who expressed an interest in using the LHF during pregnancy and/or labor were invited to take part in the study. Each woman that utilized the LHF during labor was offered the opportunity through a gatekeeper to complete the questionnaire prior to discharge from the hospital or from the community midwifery service. Informed consent to participate in the study was confirmed when the completed questionnaire was returned anonymously to the research midwife.

Before the LHF was introduced to the maternity hospital, midwives were provided with educational sessions on how to use the framework. Additional equipment such as birthing balls, birthing stools, steps, floor mats and foam blocks were also purchased for the antenatal and birthing units.

### Study-specific questionnaire

A suitable validated questionnaire was not available therefore a novel study questionnaire was designed, piloted, amended accordingly and distributed. Content validity was assessed by piloting the questionnaire among five clinicians, none of whom had any involvement in the study. This assisted in refining the logic and flow of the questionnaire, prior to its distribution to participants^18^. A pilot study of 1-month duration was conducted to refine the methodology (including data collection procedures) and to examine the reliability and validity of data collection instrument and methods. These results were excluded from the main study analysis.

The questionnaire consisted of 25 questions. Likert scales and dichotomous questions were used to obtain the quantitative data, while open-ended and free-comment questions were used to obtain more in-depth information from participants about their experiences of using the LHF. The first section of the questionnaire sought information about maternal characteristics such as age, parity, type of maternity care option attended, and if the women had attended antenatal education during their pregnancy. Specific questions relating to labor and birth outcomes included information on obstetric history (number of previous pregnancies and births), mode of birth if she had birthed before (spontaneous vaginal birth, vacuum, forceps or cesarean section, the type of pain relief used during labor, if labor had commenced naturally or was induced, and the mode of birth for the current pregnancy. Additional sections contained questions about pregnant women’s knowledge of the LHF, their perceptions of the quality and utility of the information they received, their experiences of the various steps of the LHF that they had used during labor, and the steps that were the most and least beneficial. Questions relating to various forms of pain relief used during labor and if the LHF influenced their decision-making, and choices of pain relief and confidence during labor were also included. Barriers to using each of the steps of the LHF and the perceptions of the participant’s birth partners were also included in the questionnaire and are reported elsewhere^15^.

### Outcomes of the study

Primary outcomes included steps of the LHF used by women during labor and which steps women found most helpful. Secondary outcomes included non-pharmacological pain relief (use of birthing ball, birth stool, transcutaneous electrical nerve stimulation (TENS), water therapy, hypnotherapy and homeopathy), pharmacological pain relief used during labor (entanox, pethidine and epidural analgesia) and mode of birth (spontaneous vaginal birth, assisted vaginal birth and cesarean section).

### Data analysis

Data analyses were performed using the Statistical Package for Social Science (SPSS) version 20 (SPSS Inc., Chicago, IL, USA). Frequencies and percentages were used to describe the characteristics of the participants. Associations between parity, antenatal clinic attended, and outcomes of interest were explored, using the chi-squared test for association. All reported p values are two-sided, and the level of statistical significance was set at 5%.

## RESULTS

### Characteristics of the study participants

**Figure 2:**
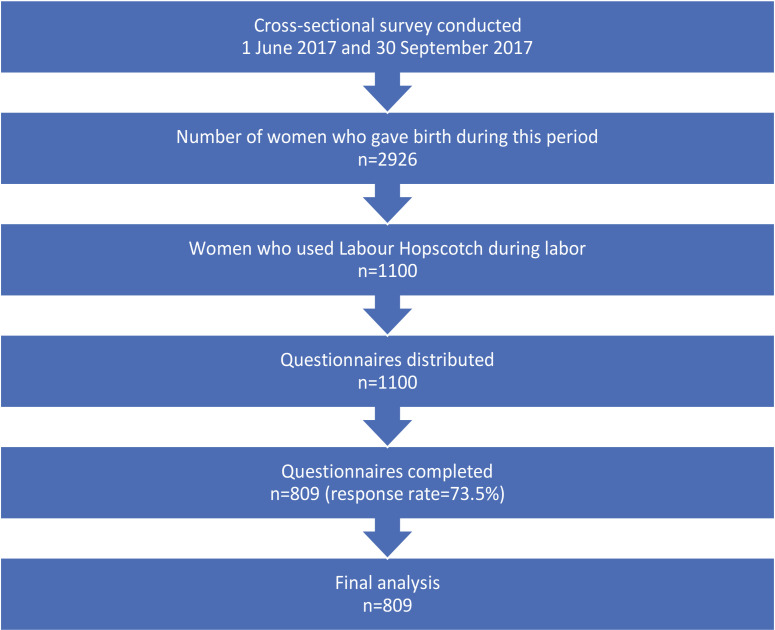
Study flowchart of sample selection

In total, 2926 women gave birth during the study period (1 June 2017 to 30 September 2017); 1100 questionnaires were distributed to women who had used the LHF and 809 women completed and returned the questionnaire, representing a response rate of 73.5% (Figure 2).

Table 1 shows the characteristics of the participants that completed the questionnaire. Nearly three-quarters of the participants (73%, n=591) were aged 30–39 years; 40% were primiparous, >60% attended obstetric-led antenatal care (public 35%, semi-private 19%, private 9%, with the remainder attending public midwifery-led services, i.e. community midwifery 26% and midwife-led clinics 11%). Fifty-eight percent of the women reported attending antenatal/labor preparation classes during their pregnancy and 3% reported having a cesarean section in a previous pregnancy.

**Table 1:** Study characteristics of participants (N=809)

*Characteristics*	*n (%)*
**Age** (years)	
18–24	40 (5.0)
25–30	154 (19.0)
31–35	364 (45.0)
36–39	227 (28.0)
≥40	24 (3.0)
**Parity**	
Primigravida	381 (47.0)
Multiparous	428 (53.0)
**Maternity care package**	
Private	74 (9.1)
Semi-private	152 (18.8)
Public	280 (34.6)
Midwife-led care	89 (11.0)
Community midwifery	214 (26.5)
Attended antenatal classes*	
Yes	467 (57.8)
No	333 (42.2)
Onset of labor**	
Spontaneous	579 (72.6)
Induced	218 (27.4)

* Missing data, n=9.

** Missing data, n=12.

Over 80% (n=657) of participants reported knowing about the LHF prior to attending the hospital in labor and had received the information primarily from midwives or community midwives during hospital antenatal classes or antenatal visits. The majority of participants reported that the information received was either excellent, very good or good. Overall, only 10% of women described the information received as fair or poor. Of these, most women (75%, n=77) had attended obstetric-led care and were less likely to know about the LHF before labor commenced. Overall, most women reported finding the LHF ‘easy to follow’ (94%, n=722/767), ‘useful’ (94%, n=715/761) and ‘helped their confidence to cope with labor’ (very confident 49%, n=374; somewhat confident 40.7%, n=310).

### Onset of labor

Nearly 73% (n=579) reported starting labor spontaneously, while 27.4% (n=218) of women reported having their labor induced.

### Pain relief used during labor

**Figure 3:**
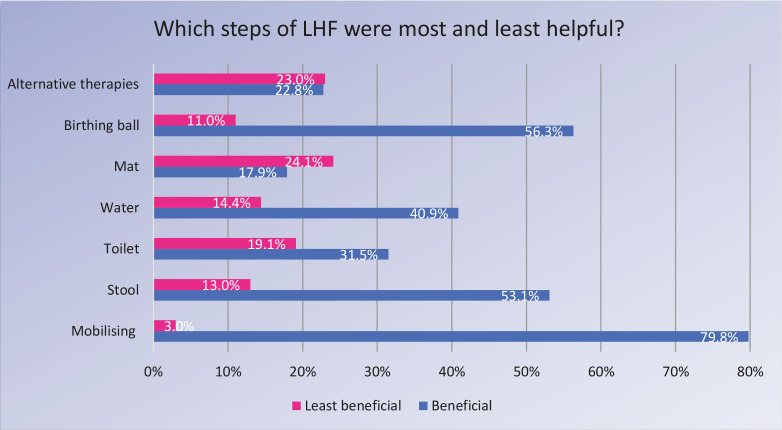
Beneficial and least beneficial steps of Labour Hopscotch

Forty percent of participants reported that the LHF had influenced their decision-making about using pharmacological pain relief in labor. When asked which of the LHF steps they found most helpful during labor, mobilizing was found to be the most beneficial by 80% (n=641) of respondents, followed by the birthing ball (56%, n=451), birthing stool (53%, n=425) and water therapy (41%, 327). The least beneficial was the birthing mat followed by alternative therapies and the toilet (Figure 3). Primiparous women were more likely to find the birthing ball (p<0.001) and birthing stool (p<0.001) beneficial during labor.

Table 2 outlines the type of non-pharmacological and pharmacological pain relief used by women during labor. Due to the lack of facilities within the hospital, the least common forms of pain relief used during labor were the birthing pool and homeopathy. Primiparous women were more likely to use non-pharmacological methods of pain relief in labor than multiparous women. In total, nearly 70% (n=545) of participants reported using entonox, and 8% (n=65) reported receiving pethidine during labor. Epidural analgesia in labor was used by 311 (38.5%) women. Primiparous women were more likely to have epidural analgesia than multiparous women (50.7%, n=193/381 vs 27.1%, n=118/428, p<0.00001).

**Table 2:** Pain relief used during labor

*Type of pain relief*	*Total (n=809) n (%)*	*Primigravida (n=381) n (%)*	*Multigravida (n=428) n (%)*	p*
**Non-pharmacological**				
Shower	215 (26.6)	125 (32.8)	90 (21.0)	**0.0002**
Pool	12 (1.5)	3 (0.8)	9 (2.1)	0.14
Tens machine	157 (19.4)	98 (25.7)	59 (13.8)	**<0.0001**
Hypnobirthing	79 (9.8)	45 (11.8)	34 (7.9)	0.07
Homeopathy	37 (4.6)	24 (6.3)	13 (3.0)	**0.03**
Other	164 (20.3)	89 (23.4)	75 (17.5)	**0.04**
None	94 (11.6)	23 (6.0)	71 (16.6)	**<0.00001**
**Pharmacological**				
Entanox	545 (67.5)	277 (72.7)	268 (62.6)	**0.002**
Pethidine	65 (8.0)	49 (12.9)	16 (3.7)	**<0.00001**
Epidural	311 (38.5)	193 (50.7)	118 (27.6)	**<0.00001**

* Significant at p<0.05.

The use of epidural analgesia during labor (Table 3) was also more common in women who had attended either private (p=0.004) or public-led obstetric (p=0.005) antenatal care. Women who had attended the community midwives antenatally were least likely to have an epidural during labor (p<0.00001).

**Table 3:** Characteristics of women with and without use of epidural analgesia during labor

*Characteristic/Outcome*	*No Epidural*		*Epidural*		
	*n=498*		*n=311*		
	*n*	*(%)*	*n*	*(%)*	p*
**Age** (years)					
18–24	21	(4.2)	19	(6.1)	0.23
25–30	91	(18.3)	63	(20.3)	0.48
31–35	224	(45.0)	140	(45.0)	0.99
36–39	149	(29.9)	78	(25.1)	0.14
≥40	13	(2.6)	11	(3.5)	0.45
**Parity**					
Primigravida (P1)	188	(37.8)	193	(62.1)	**<0.00001**
Para 2	211	(42.4)	80	(25.7)	**<0.00001**
Para 3 or more	91	(18.3)	38	(12.2)	**0.02**
**Antenatal care clinic**					
Private obstetric	34	(6.8)	40	(12.9)	**0.004**
Semi-private obstetric	84	(16.9)	68	(21.9)	0.08
Public obstetric	154	(30.9)	126	(40.5)	**0.005**
Midwives’ clinic	55	(11.0)	34	(10.9)	0.96
Community midwifery	171	(34.3)	43	(13.8)	**<0.00001**
**Mode of birth**					
Spontaneous vaginal birth	430	(86.3)	194	(62.4)	**<0.00001**
Assisted vaginal birth	27	(5.4)	88	(28.3)	**<0.00001**
Cesarean section	41	(8.2)	29	(9.3)	0.59

* Significant at p<0.05.

### Mode of birth

Overall, the spontaneous vaginal birth (n=594/809), assisted birth (n=115/809) and cesarean birth rate (n=70/808) were 77.1%, 14% and 8.7%, respectively. Significantly higher rates of spontaneous vaginal birth were achieved in women who did not have epidural analgesia during labor (p<0.00001). Women who had epidural analgesia were significantly more likely to have an assisted birth (28.3% vs 5.4%; p<0.00001). The rate of cesarean section births was not significantly different between the women who were or were not administered epidural analgesia during labor as shown in Table 3.

## DISCUSSION

The Labour Hopscotch Framework is a midwife-led innovation developed and implemented to empower women to keep active during labor and assist midwives in facilitating physiological labor and birth. The aim of this study was to evaluate which steps of the LHF women found most useful, the use of non-pharmacological and pharmacological methods of pain relief used during labor and finally to investigate the labor and birth outcomes of women who used LHF during labor.

The age and parity of participants in our study reflects the national and international trend over the last decade where the rate of first-time mothers over the age of 30 years has increased^19^. Hospital and national data on choice of maternity care are not publicly available in Ireland, however when we compared our findings with another Irish study conducted between 2012 and 2017^20^, the rate of women attending public maternity care (obstetric- and midwifery-led) were higher (72% vs 61%), while the rate of women attending semi-private or private care was lower (28% vs 38%). The rate of induction of labor was similar to the general population in the study site (29.4%) and is also consistent with Irish rates of induction of labor which ranged from 19.7% to 35.8% across maternity units in Ireland in 2017^21^.

Fifty-eight percent of women in our study reported attending antenatal classes during their pregnancy. Women also reported that the LHF was a useful preparation tool for labor and birth, providing structure and helped them to focus. Several participants have previously reported that they used it before labor to visualize the natural labor process, to plan and practice steps or positions in advance, and even to proactively induce the labor by themselves in certain situations (e.g. SROM and postdates)^15^. It is possible that LHF motivated women to take initiative and play an active role in their preparation for and during labor. Randomized controlled trials (RCTs) have found that women who receive antenatal education are more likely to arrive at the hospital in active labor, are less likely to be induced, use less analgesia and have higher vaginal birth rates^22-24^. A recent systematic review and meta-analysis^25^ also found an association between attendance at antenatal classes and decreased anxiety levels and increased self-sufficiency levels. Decreased anxiety levels result in lower levels of adrenaline, increasing endogenous oxytocin release, resulting in efficient uterine contractions and therefore promoting progress in labor.

The most commonly steps of LHF used by women in our study were mobilizing, the birthing ball, and birthing stool. Positioning during labor influences the characteristics and effectiveness of uterine contractions, fetal well-being, maternal comfort, and subsequent course of labor. The steps of the LHF encourage movement, gravity and upright positions during the antenatal period and during labor. Mobilization during labor enables gravity, thus assisting the descent of the fetal head directly onto the woman’s cervix, further intensifying uterine contractions. Lawrence et al.^11^ explored the impact of the use of positions and activity on the duration of the first stage of labor and subsequent mode of birth. Sixteen RCT studies examined the effects of upright positioning (sitting, standing, walking, and kneeling) compared to recumbent positions. Women randomized to upright positions had a statistically significant reduction in the duration of first stage of labor by approximately one hour, in comparison to supine and recumbent positions (9 trials, mean difference=0.99; 95% CI: -1.60 – -0.39). Women were also less likely to request epidural analgesia (RR=0.83; 95% CI: 0.72–0.96, p=0.01). A significant number of women in our study also used the birthing ball before and during labor. Sitting upright on a birthing ball enables the woman to further incorporate movement, leaning forward, pelvic rocking, swaying, figure-of-eight hip circles or gently bouncing. These movements enable descent of the presenting part to become well applied to the woman’s cervix, uterine contractions are intensified in strength, regularity, and frequency. Efficient uterine contractions subsequently aid cervical dilatation. In addition, a woman’s position on the birthing ball can help relieve pressure and tension of the lower back, and are thus associated with shorter labor and reduced need for epidural analgesia^26,27^. Being able to choose positions can also increase women’s experience of ‘being in control’^28^, a major factor contributing to a positive birth experience^29^.

The most commonly used pharmacological methods of pain relief in our study were entanox and epidural analgesia. One of the aims of our study was to examine the rate of epidural analgesia in women who used the steps of the LHF. We note that a high proportion of primiparous women (62.1%) received an epidural during labor and this requires further exploration. Active management of labor is a philosophy of care that is practiced within the hospital, it is possible that some primigravida may have had an amniotomy on admission to labor and oxytocin to accelerate labor if poor progress was diagnosed (<1 cm/h over 2 hours). It may also be associated with the wide availability and access of epidural analgesia in the hospital site. Notably though, since the full implementation of the LHF to the study site, the overall hospital epidural analgesia rate has continued to fall year-on-year, from 57% in 2017 to 52% in 2018, and 50.1% in 2020.

The spontaneous vaginal birth rate in women using the LHF was 77.1%, higher than the overall hospital rate (59.8%) and the national rate (53.8%). Previously women have reported that the LHF supported them psychologically, empowering them to stay at home for longer in the early stages of labor^15^. Admission in the latent phase of labor is associated with higher intrapartum interventions such as cesarean section or an instrumental birth, artificial rupture of membranes, oxytocin augmentation, and epidural analgesia^30-32^. These findings could also be attributed to women’s increased activity and mobility during labor, ability to cope and progress in labor, therefore disrupting the cycle of intervention and promoting physiological labor and birth. It is also conceivable that the results could have been influenced by the enthusiasm and motivation of midwives and obstetricians. Personal preferences and attitudes of midwives and obstetricians are known to play an important role in supporting women to achieve physiological labor and birth^9,33-35^.

The overall rate of assisted vaginal birth was similar to the hospital and national rate. The higher rates of assisted vaginal birth in women who had epidural analgesia during labor are consistent with the findings of other studies^3^. The cesarean section rate of women who used LHF during labor was 8.7%. This is considerably lower than the overall hospital rate (27.2%) and the national average (31.3%).

Significant changes are taking place in the provision of Irish maternity services. There is a move towards an integrated model of care, where midwives will be the lead healthcare professional for women defined as ‘normal risk’^36^. To successfully support the implementation of this model of care going forward, it is imperative that midwives and midwifery students are educated to be competent and confident in employing strategies that support physiological childbirth for the women in their care. Our research findings, core concepts and clinical skills associated with the LHF are currently embedded in the undergraduate and postgraduate midwifery curricula of students affiliated with the study hospital site. It is also essential that midwifery students have opportunities to participate in physiological care practices and observe how midwives advocate for physiological birth in their interactions with women so that they too will carry these midwifery skills into their future practice.

### Strengths and limitations

It is important to state that the results presented represent one large maternity unit in Ireland and may not be generalizable to settings with differing models of maternity care and availability of non-pharmacological and pharmacological methods of pain relief. There was very limited availability of birthing pool and homeopathy during the period of this study. Almost all questionnaires were completed by women before being discharged home (usually within 24 to 48 hours) therefore reducing the likelihood of recall bias. It is important, however, to note that people may provide more socially desirable responses by completing a questionnaire in the proximity of healthcare providers^37^ making the responses obtained from the participants more vulnerable to the bias of accommodating social expectations. A consideration for future studies would be to survey women after they leave their healthcare environment.

Since the completion of this study, the Health Service Executive has supported a national roll-out of the Labour Hopscotch Framework to all nineteen maternity units in the Republic of Ireland, which began in 2020. At the time of writing, the LHF has been implemented in nine birthing units across Ireland. Plans are also in place to incorporate the LHF during the implementation of the National Standards for Antenatal Education^38^. Extending this study nationally to include all maternity units and alternative birth settings in Ireland will increase transferability. Maternal characteristics and obstetric variables such as ethnicity, BMI, cervical dilatation on admission, length of the first and second stage of labor, oxytocin use, birthweight and head circumference are also important confounders when assessing the association between pain relief used in labor and mode of birth, and should be considered in future studies.

## CONCLUSIONS

Internationally, the philosophy of midwifery is underpinned by an assumption that maternity care should be woman centered, and promotes and protects physiological birth. The implementation of the Labour Hopscotch Framework enables midwives to fulfil this philosophy. Our study findings contribute to the increasing national and international evidence that midwifery-led initiatives and practices such as LHF can promote and advocate for women to be active and mobile during labor and thus support physiological birth.

## Data Availability

The data supporting this research are available from the authors on reasonable request.
